# Supplementary material for: Ca-dimers, solvent layering, and dominant electrochemically active species in Ca(BH4)2 in THF
Source: Nat Commun. 2024 Feb 15;15:1397. doi: 10.1038/s41467-024-45672-7 (PMC11258298; doi:10.1038/s41467-024-45672-7)
Supplement: Supplementary file 1 — Supplementary Information [file 41467_2024_45672_MOESM1_ESM.pdf]

Supplementary Information: Ca-dimers, solvent layering, and dominant electrochemically active species in $Ca(BH_4)_2$ in THF

Ana Sanz-Matias,[1,2] Fabrice Roncoroni,[1,2] Siddharth Sundararaman,[1,2] and David Prendergast[1,2,*]

[1] *Joint Center for Energy Storage Research, Lawrence Berkeley National Laboratory, Berkeley, CA 94720, USA*
[2] *The Molecular Foundry, Lawrence Berkeley National Laboratory, Berkeley, CA 94720, USA*

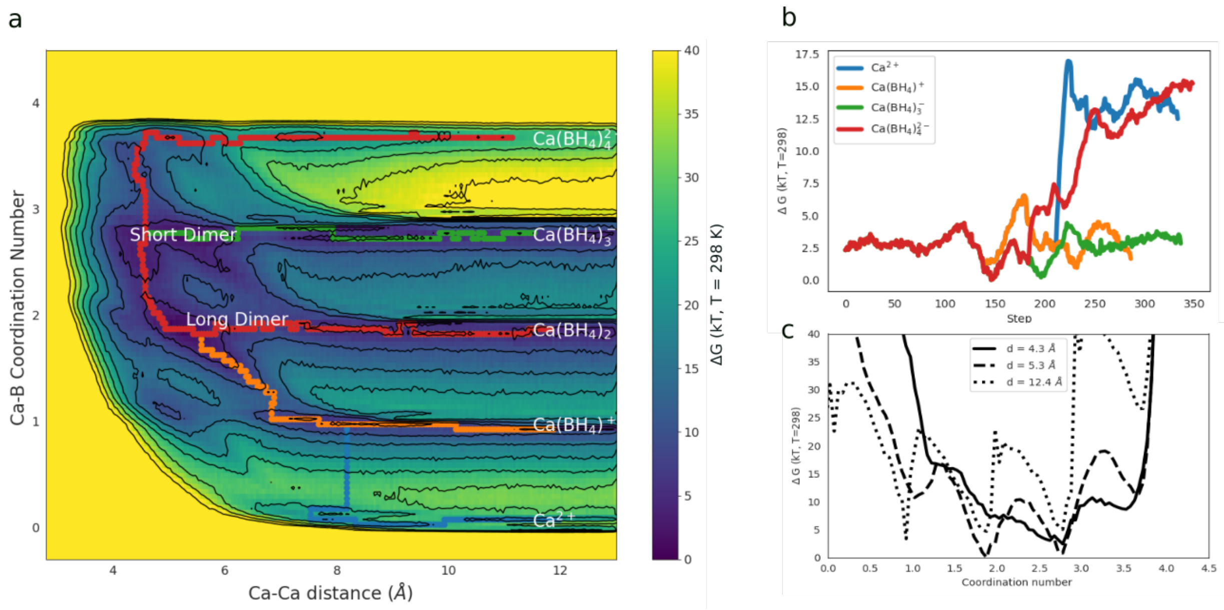

FIG. S1: (a) Bulk free-energy surface of dimerization of ionic and neutral species, controlled by borohydride coordination number. (b) Free energy profiles for bulk disproportionation pathways with colors corresponding to the same indicated on the free-energy surface in (a). (c) Direct dissociation pathways by removal of borohydride obtained from vertical slices through the free-energy surface at specific Ca-Ca separations.

* dgprendergast@lbl.gov

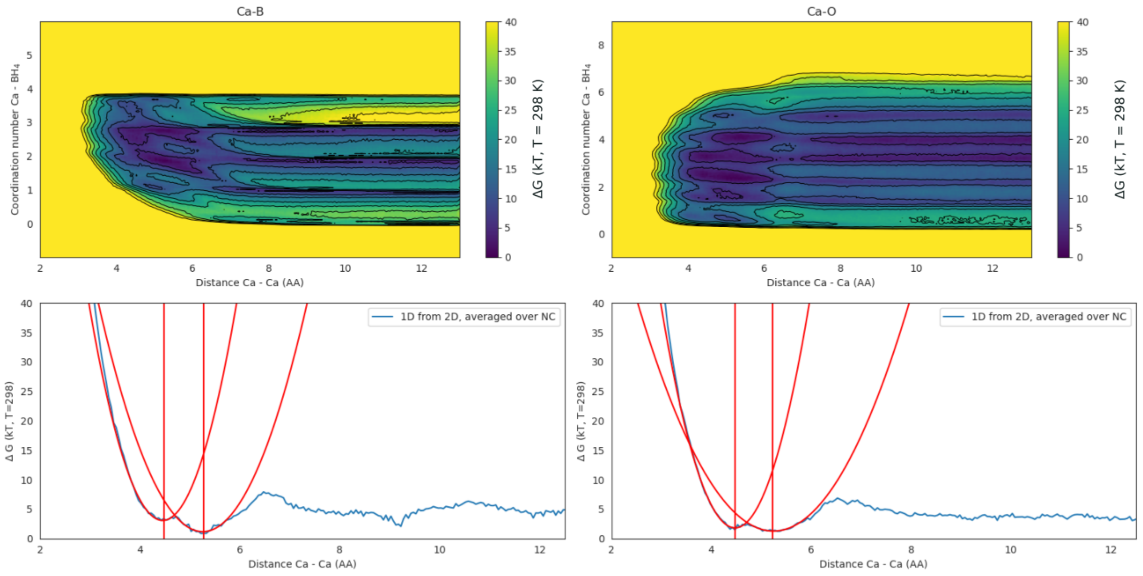

FIG. S2: **Determination of short and long dimers equilibrium RT distances.** Free-energy of dimerization (distance Ca-Ca) as a function of the Ca-B(H_4) coordination number (left) and Ca-O(THF) coordination number (r_0 = 3.6 Å, right). One-dimensional profiles obtained through thermodynamic integration over each coordination number allow us to interpolate the equilibrium distances of the short and long dimer.

Fig.	THF	Ca^{2+}	BH_4^-	G	Box pars. (Å)	CVs	q_G (e^-)	W	H	Freq	Reps.	RepFreq	Time (ns)
Bulk	200	2	4	-	30.3 x 30.3 x30.3	dCa [0:16] Å	-	0.05	0.02	200	20	50000	1660
1						CN(Ca-B) [0-4]		0.05					
Neutral<br>2,3,<br>5	336	2	4	2	33.9 x 31.8 x 49.8	dCa [0:16] Å<br>CN(Ca-B) [0-4]<br>dZ [0:25]	G1 = G2 = 0	0.2<br>0.2<br>0.5	0.02	200	70	50000	2082
2	336	-	-	2	33.9 x 31.8 x 49.8	dZ [0:25] Å	G1 = G2 = 0	0.05	0.02	200	15	50000	1049
2	336	-	-	2	33.9 x 31.8 x 49.8	dZ [0:25]	G1 = -1.4; G2 = 0	0.05	0.02	200	10	50000	620
CS1	514	2	4	2	33.9 x 31.8 x 71.8	CN(Ca-Ca) [0:1] Å<br>CN(Ca-B) [0-4]<br>dZ [2:70]	G1 = -0.7;<br>G2 = +0.7	0.2<br>0.2<br>0.25	0.04	200	70	50000	1438
CS2<br>2,4,<br>5	514	2	4	2	33.9 x 31.8 x 71.8	CN(Ca-Ca) [0:1] Å<br>CN(Ca-B) [0-4]<br>dZ [2:70]	G1 = -1.4;<br>G2 = +1.4	0.2<br>0.2<br>0.25	0.04	200	70	50000	1464

TABLE S1: **Metadynamics simulation details.** Figure number or label, box composition (Ca^{2+}, BH_4^- and graphene) and equilibrated lattice parameters, collective variables and their ranges, surface charges, and metadynamics parameters (hill width and height, frequency, number of replicas, replica update frequency, and total simulation time).

Supplementary Information 1. DIPOLE MOMENTS OF CA(BH_4)$_2$ ISOMERS

In order to compute the dipole moment of specific $Ca(BH_4)_2$ isomer, an average structure of the whole group was calculated (including THF solvating molecules). Then, using Density Functional Theory as implemented in Terachem,[1] the atomic positions were optimized to obtain the dipole moment. Typically, a decrease in the dipole moment of about 1 D is observed upon minimization. The B3LYP[2] exchange-correlation functional with a dispersion correction (D3)[3] and a 6-311G basis set were used.

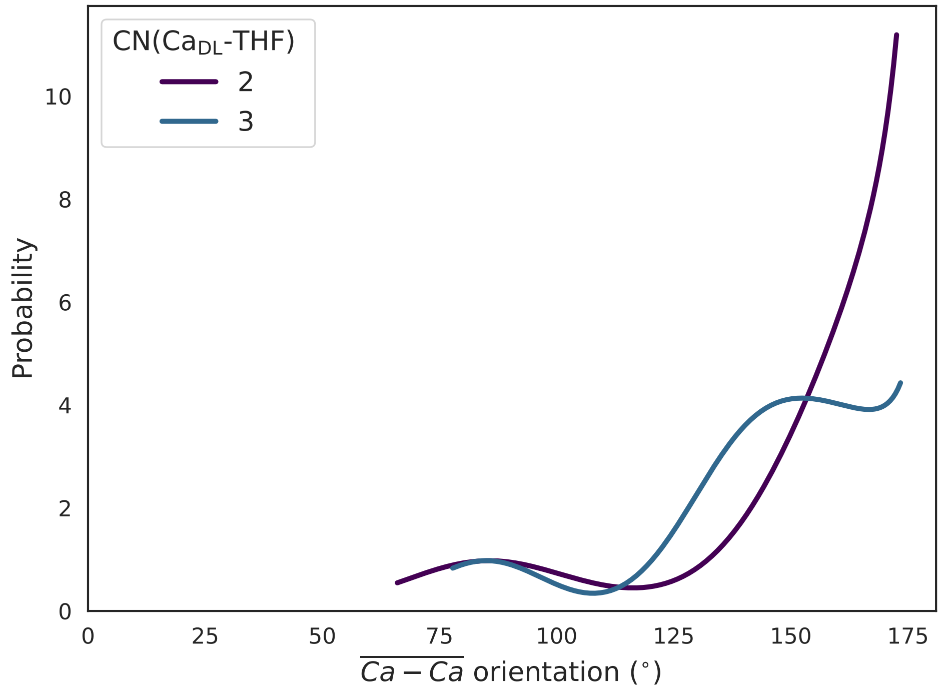

FIG. S3: **Solvent coordination of perpendicular dimers.** Orientation of the Ca-Ca axis with respect to the surface normal of structures of the isomer SD 5 THF - 4 at the DL (see Fig. 3 in the main text) depending on the THF-Ca coordination of the Ca that resides in the DL. At strongly perpendicular orientations (larger that 150 ° with respect to the surface normal), two-fold solvent coordination is favored.

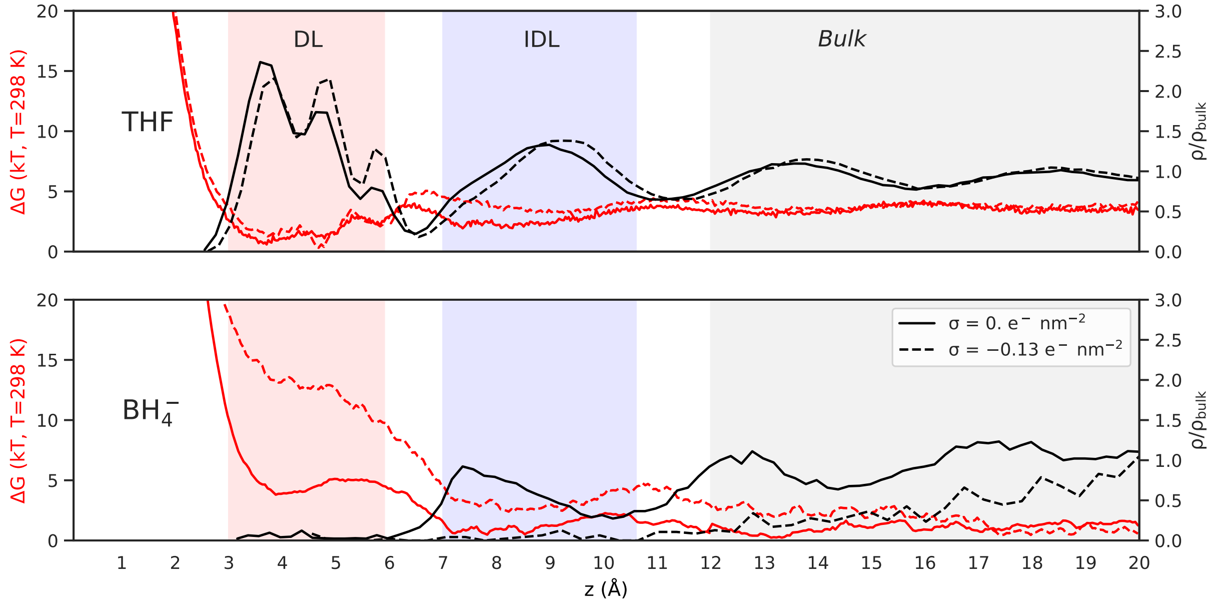

FIG. S4: **Free-energy of BH_4^- at the (biased) interface.** The free-energy profile of a single THF molecule in THF (top) at RT with respect to distance from a graphite interface (red) and the corresponding oxygen density profile (black), with slight adjustment (dashed lines) at negative bias. The Dense Layer (DL), Intermediate Density Layer (IDL) and bulk regions are color-coded hereafter in red, blue and gray. The bottom figure shows the analogous free-energy profile of a single BH_4^- molecule in THF at RT with respect to distance from a graphite interface (red) and the corresponding boron density profile (black).

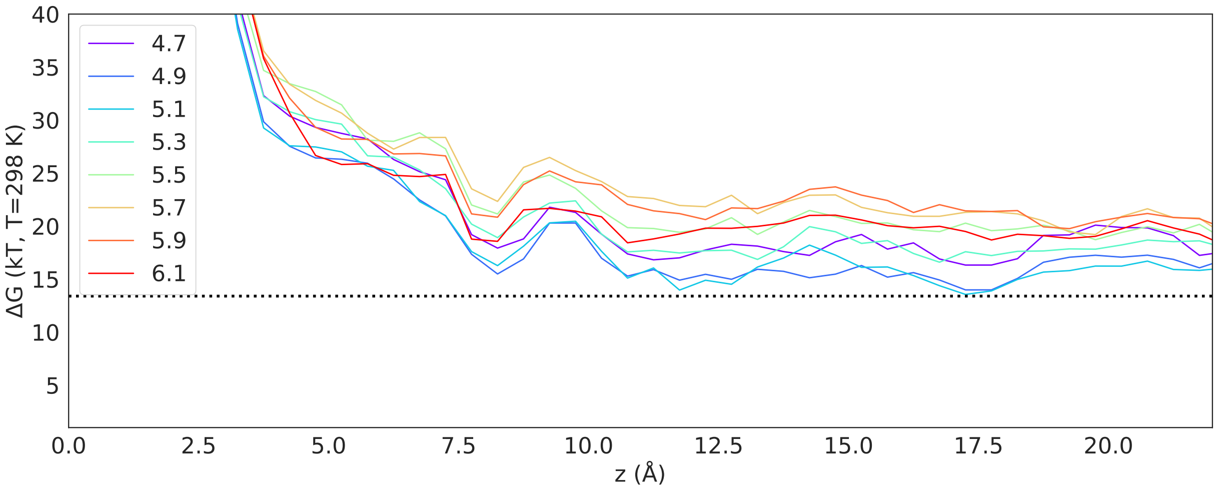

FIG. S5: **Interfacial free-energy of [1,4] dimers.** Free energy pathways as a function of distance from the neutral surface for dimers with [1,4] Ca-B$(H_4)^-$ coordination numbers at different Ca-Ca distances. The most favorable, with d(Ca-Ca) = 4.9-5.1 Å, preferably sits far away from the layered interface, at ~ 17.5 Å.

Supplementary Information 2. REDUCTION POTENTIALS

We have estimated the onset potential for reduction, $\mathcal{E}$, as follows:

$$\begin{aligned} \mathcal{E} <\ & \mathcal{E}^0 + [\ln n_O(z)dV + \mu_O^{\text{excess}}(z)]/e \\ & +\phi(z) + [\Phi_O(z) - \Phi_R(z)]/e \end{aligned} \tag{S1}$$

where $\mathcal{E}^0 = -\Delta G^{\text{red}}/nF$, the standard reduction potential, is approximated as $-[E_{\text{solv}}[R] - E_{\text{solv}}[O] - \epsilon_F]/nF$ for an n-electron process (F is Faraday's constant, ϵ_F is the metal electrode work function). In this work we focus on single-electron transfer events only (n=1) for electrolyte species. This term was obtained individually for each isomer structure using Density Functional Theory (we used the QCHEM code[4] through the PyQchem interface.[5] The B3LYP[2] exchange-correlation functional with a dispersion correction (D3)[3] and a 6-311G basis set were used, with implicit solvation modeled using PCM.[6] The activity-dependence in this expression is captured by two terms related to: (1) the concentration profile of each species before reduction, $n_O(z)$ (dV is the smallest volume element in our continuum model, set equal to the effective volume of the smallest species); and (2) the excess chemical potential, $\mu_O^{excess}(z)$, which includes non-ideal concentration contributions mostly due to volume exclusion. The electrostatic potential profile is indicated by $\phi(z)$, as function of distance from the electrode. These profiles were obtained self-consistently with our continuum model, employing as inputs the specific adsorption potential profiles of the oxidized and reduced species, $\Phi_O(z)$ and $\Phi_R(z)$, which were computed with molecular-dynamics-based free-energy sampling in the dilute limit.

Supplementary Information 3. THERMODYNAMIC REDUCTION POTENTIAL

In order to estimate the overpotential, we computed the thermodynamic reduction potential of the $2e^-$-reduction-deposition process of a dication in the bulk electrolyte, with reference to Ca^{2+} in vacuum so as to enable comparisons with the interfacial reduction potentials, as follows:

$$\Delta G^{red-dep} = \Delta G^{red}_{(g)} - \Delta G_{(sol)} - E_{at} - n(\epsilon_F - \varphi(0)) \tag{S2}$$

Then, the potential at the electrode required for thermodynamically favorable reduction-deposition ($\Delta G^{red-dep} \leq 0$) can then be defined as:

$$\varphi(0)^* \leq \epsilon_F - (\Delta G^{red}_{(g)} - \Delta G_{(sol)} - E_{at})/n \tag{S3}$$

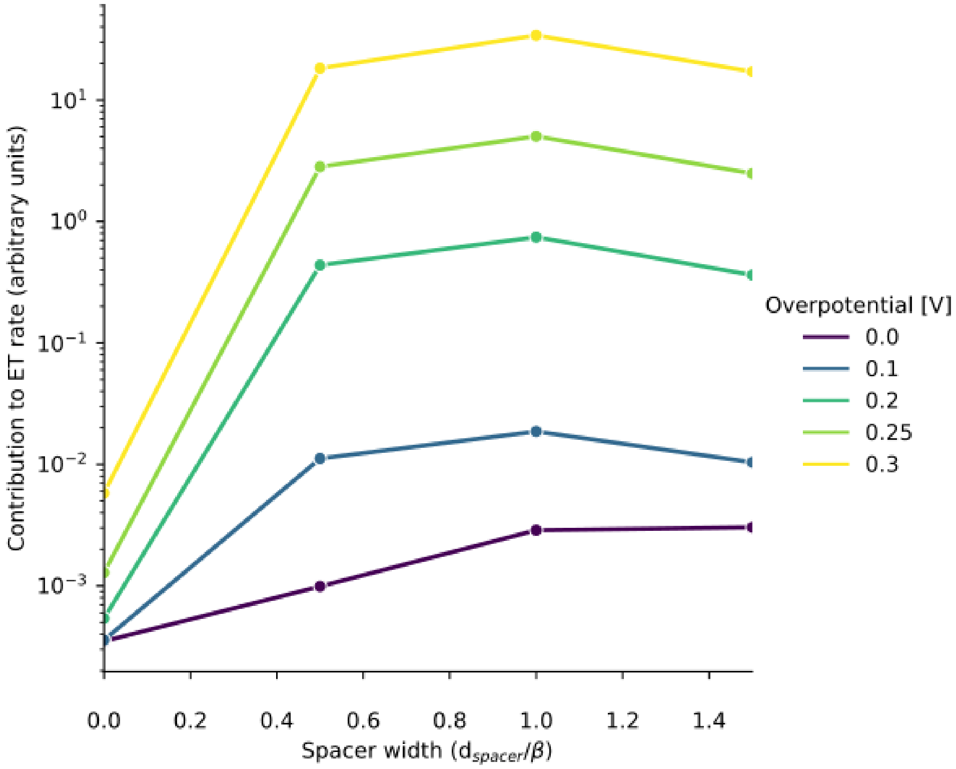

FIG. S6: Contributions to the electron transfer rate integrated throughout the interface for various overpotentials as a function of the dielectric spacer width (expressed as a fraction of the tunneling decay length z_0 at 0.12 M.

We assume a calcium metal electrode, with Fermi energy ϵ_F = -2.84 V, calcium atomization energy E_{at} = 1.845 V,[7] and n=2 (averaging over two electron transfer steps). We obtain $\Delta G^{red}_{(g)}$ and $\Delta G_{(sol)}$ from DFT calculations on $Ca^{2+}(THF)_6$ isomers favored in the bulk (see Fig.S7, bottom right). A thermodynamic average of the values of φ (0)* for each isomer using bulk populations yields a bulk thermodynamic reduction potential for Ca^{2+} in THF of -2.25 V.

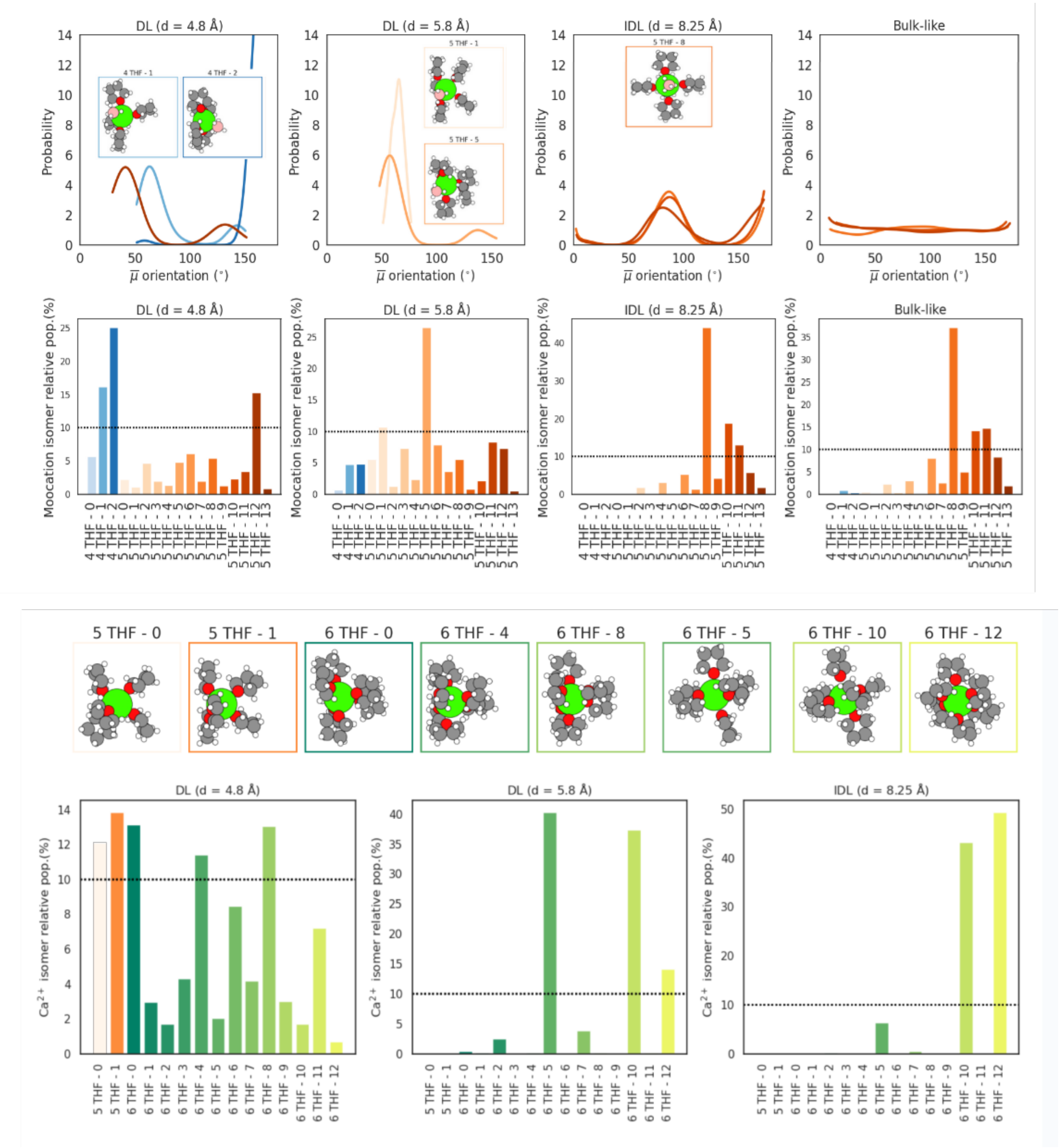

FIG. S7: Population distribution of monocation (top) and dication (bottom) isomers by a biased electrode (CS2). Whole solvent and BH_4 molecules are included in the coordination sphere. For the monocation, dipole orientation distribution of isomers with population larger than 10% per layer are shown at the top. The angle is calculated with respect to the surface normal, with 180 being the BH_4 pointing away from the electrode. The bottom histograms show isomer populations obtained from unsupervised learning analysis close to the center of the DL (z = 4.8 Å), close to the edge of the DL (z = 5.8 Å) , in the IDL (z = 8.25 Å), and in the bulk. Representative atomic structures of the dominating isomers are shown in the inset in their preferred orientation, with the surface on the left. Note the similar population distributions in the bulk and IDL, and the 'flattened' DL isomers for monocations (4 THF - 1, 4 THF - 2, 5 THF - 1, and 5 THF - 5) and dications (5 THF - 0 and 1, and 6 THF - 0, 4 and 8).

REFERENCES

[S1] Ufimtsev, I. S. & Martinez, T. J. Quantum chemistry on graphical processing units. 1. strategies for two-electron integral evaluation. *Journal of Chemical Theory and Computation* **4**, 222–231 (2008).

[S2] Stephens, P. J., Devlin, F. J., Chabalowski, C. F. & Frisch, M. J. Ab initio calculation of vibrational absorption and circular dichroism spectra using density functional force fields. *The Journal of Physical Chemistry* **98**, 11623–11627 (1994).

[S3] Grimme, S., Antony, J., Ehrlich, S. & Krieg, H. A consistent and accurate ab initio parametrization of density functional dispersion correction (dft-d) for the 94 elements h-pu. *The Journal of Chemical Physics* **132**, 154104 (2010).

[S4] Shao, Y. *et al.* Advances in molecular quantum chemistry contained in the q-chem 4 program package. *Molecular Physics* **113**, 184–215 (2015).

[S5] Carreras, A. & Casanova, D. (2023). URL `https://pyqchem.readthedocs.io/en/master/`.

[S6] J. Tomasi, B. M. & Cancès, E. The ief version of the pcm solvation method: An overview of a new method addressed to study molecular solutes at the qm ab initio level. *Molecular Physics* **464**, 211–226 (1999).

[S7] Oxtoby, D. W., Gillis, H. P. & Nachtrieb, N. H. *Principles of modern chemistry* (Thomson/Brooks/Cole [Pacific Grove, CA], [Pacific Grove, CA], c2002).
